# Cortico-amygdala interaction determines the insular cortical neurons involved in taste memory retrieval

**DOI:** 10.1186/s13041-020-00646-w

**Published:** 2020-07-28

**Authors:** Konami Abe, Marin Kuroda, Yosuke Narumi, Yuki Kobayashi, Shigeyoshi Itohara, Teiichi Furuichi, Yoshitake Sano

**Affiliations:** 1 Department of Applied Biological Science, Tokyo University of Science, Noda, Chiba 278-8510 Japan; 2 Laboratory for Behavioral Genetics, Center for Brain Science, Wako, Saitama 351-0198 Japan; 3 Present Address: Brain/MINDS, RIKEN Center for Brain Science, Wako, Saitama 351-0198 Japan

**Keywords:** Memory allocation, Insular cortex, Basolateral amygdala, Conditioned taste aversion, Taste memory, Functional interaction, Structural interaction

## Abstract

The insular cortex (IC) is the primary gustatory cortex, and it is a critical structure for encoding and retrieving the conditioned taste aversion (CTA) memory. In the CTA, consumption of an appetitive tastant is associated with aversive experience such as visceral malaise, which results in avoidance of consuming a learned tastant. Previously, we showed that levels of the cyclic-AMP-response-element-binding protein (CREB) determine the insular cortical neurons that proceed to encode a conditioned taste memory. In the amygdala and hippocampus, it is shown that CREB and neuronal activity regulate memory allocation and the neuronal mechanism that determines the specific neurons in a neural network that will store a given memory. However, cellular mechanism of memory allocation in the insular cortex is not fully understood. In the current study, we manipulated the neuronal activity in a subset of insular cortical and/or basolateral amygdala (BLA) neurons in mice, at the time of learning; for this purpose, we used an hM3Dq designer receptor exclusively activated by a designer drug system (DREADD). Subsequently, we examined whether the neuronal population whose activity is increased during learning, is reactivated by memory retrieval, using the expression of immediate early gene c-fos. When an hM3Dq receptor was activated only in a subset of IC neurons, c-fos expression following memory retrieval was not significantly observed in hM3Dq-positive neurons. Interestingly, the probability of c-fos expression in hM3Dq-positive IC neurons after retrieval was significantly increased when the IC and BLA were co-activated during conditioning. Our findings suggest that functional interactions between the IC and BLA regulates CTA memory allocation in the insular cortex, which shed light on understanding the mechanism of memory allocation regulated by interaction between relevant brain areas.

## Introduction

Taste memory is critically important for animals to evaluate the safety and nutritional value of foods. Conditioned taste aversion (CTA) is a robust associative learning in which animals associate a novel taste (such as saccharine; conditioned stimulus [CS]) with an aversive experience (such as malaise, unconditioned stimulus [US]), which leads to the shift of the valence of tastant to aversion. The insular cortex (IC) processes multisensory information, including gustatory [1–3] and visceral [4, 5] modalities. Plasticity related gene expression and protein synthesis in the IC is necessary for CTA memory formation [6–9]. The inactivation of the IC leads to the impairments of acquisition and suppression of CTA memory retrieval [10–14]. The basolateral amygdala (BLA) is another important brain area for CTA memory formation [11, 15, 16]. The IC and BLA are reciprocally connected, and these interconnections are important for the retention of CTA memory [17]. It has also been shown that high frequency stimulation of the BLA induces long-term potentiation (LTP) in the IC, and the induction of LTP in the BLA-IC pathway before CTA training increases the retention of memory [18–22]. In addition, CTA training induces subsequent LTP in the BLA-IC pathway and increases amygdala-cortical function connectivity [23, 24]. Recently, it was shown that IC neurons projecting to the BLA is involved in the CTA memory formation and retrieval [25, 26]. However, the mechanism by which a neuronal population encoding CTA memory in the IC is determined is not fully understood.

CTA learning activates a subpopulation of IC neurons [27]. The cyclic-AMP-response-element-binding protein (CREB) is a transcription factor that regulates memory formation. Previously, we showed that CREB levels determine the IC neurons that proceed to encode a given conditioned taste memory [28]. CREB commonly regulates memory allocation in the amygdala and hippocampus for fear memory association [29–34] and in the retrosplenial cortex for spatial information [35]. Electrophysiological studies indicated that amygdala neurons with higher CREB levels are more excitable [31, 36]. Modulation of neuronal activity using a designer receptor, the modified human muscarinic acetylcholine receptor type 3 (hM3Dq), exclusively activated by a designer drug (DREADD) and step function opsin (SFO) in a random population of amygdala neurons determines the neurons that are incorporated into a fear memory trace [33, 37]. In the hippocampus, it was also shown that cellular activity regulates spatial memory allocation and linking of two distinct memories by sharing neural ensemble [38].

Although cellular mechanism of memory allocation is well studied in the amygdala, the mechanism in IC has not been addressed yet. Furthermore, regulation of memory allocation by the interaction between relevant brain areas is not well known. CTA is an ideal model to address this issue because 1) the IC and BLA are reciprocally connected; 2) they are necessary for CTA learning; and 3) taste memory is allocated to a subset of neurons in the IC and BLA. In this study, we manipulated neuronal activity during CTA conditioning in a subset of IC and/or BLA neurons expressing hM3Dq receptors and analyzed whether these pre-activated neurons were preferentially activated by memory retrieval, based on the expression of c-fos protein. We also investigated the afferent and efferent IC neuronal activation by using c-fos expression coupled with retrograde and anterograde transsynaptic tracers.

## Results

### IC neurons activated by memory retrieval do not depend on neural activity during learning

**Fig. 1:**
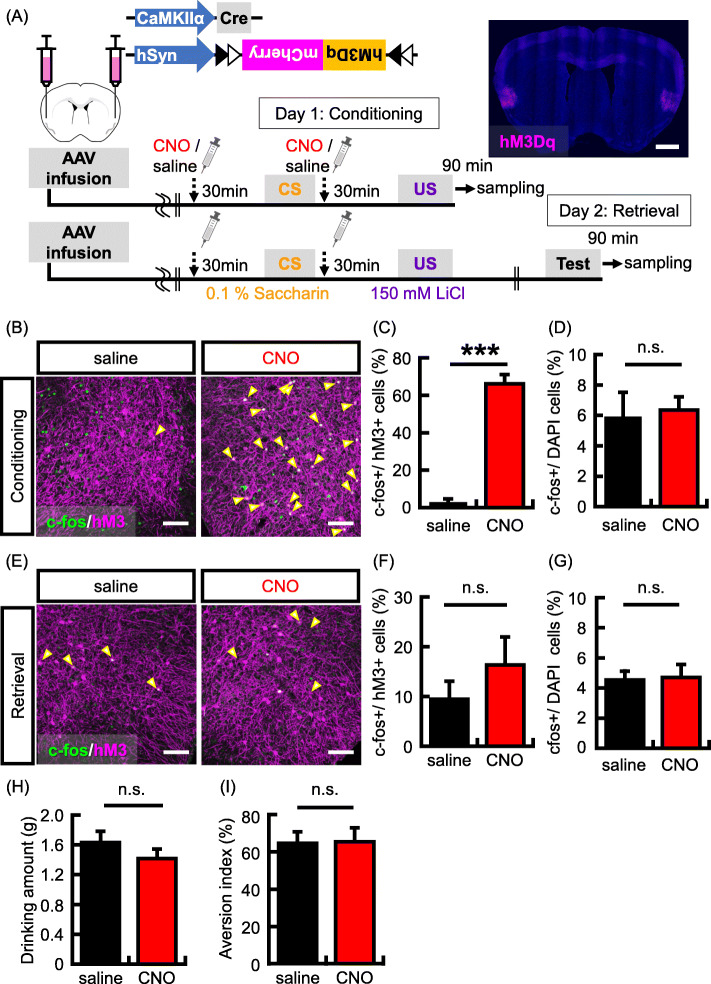
Increasing neuronal activity in a subset of IC neurons during CTA conditioning. **a** The top panel shows the map of the AAV constructs. Bottom panel shows experimental schema. Representative picture showing localized hM3Dq (magenta) expression in the IC by hM3Dq-mCherry virus infection. Scale bar = 1000 um. **b** Representative images showing expression of c-fos (green) following conditioning and hM3Dq (magenta). Yellow arrows indicate double-labeled neurons (c-fos + and hM3Dq+). Scale bar = 100 um. (C-D) Probability of expression of c-fos (c-fos+) in cells transduced with the hM3Dq viruses (hM3Dq + neurons) (**c**) and stained with DAPI (DAPI+ cells) (**d**) in the IC (*N* = 3 in each group). **e** Representative images showing expression of c-fos (green) following memory retrieval test and hM3Dq (magenta). Scale bar = 100 um. **f**-**g** Probability of occurrence of c-fos + neurons in the hM3Dq + neurons (**f**) and DAPI+ cells (**g**) in the IC (*N* = 7 in each group). **h** Data show the mean quantity of saccharine solution consumed during the conditioning. **i** Data showing the aversion index in the memory retrieval test (*N* = 13 in each group). Data are presented as mean ± SEM; ***p < 0.001 (saline-injected mice, black columns; CNO-injected mice, red columns)

We have previously shown that CREB levels determine which the IC neurons that proceed to encode a given conditioned taste memory [28]. However, the cellular mechanism of memory allocation in IC is unclear. We as well as other researchers showed that excitable neurons are preferentially involved in the process of memory formation in the amygdala, and these neurons are reactivated during memory retrieval [33, 37]. In the previous study, hM3Dq, a Gq protein coupled receptor- was used to manipulate neuronal activity in the amygdala neurons [37]. The hM3Dq showed no constitutive activity in increasing neuronal activity [39]. A synthetic ligand, clozapine-N-oxide (CNO), but not an endogenous ligands for muscarinic receptors can selectively bind to and activate the hM3Dq receptors [40]. The activation of amygdala neurons expressing hM3Dq during auditory fear conditioning determined the neurons that are involved in fear memory retrieval [37]. In this study, hM3Dq was expressed in a subset of IC neurons to induce neuronal competition; we also tested whether the activation of IC neurons by hM3Dq enhances the process of memory allocation as in the amygdala (Fig. 1a). To express hM3Dq in a subset of IC neurons, we utilized the Cre-dependent hM3Dq expression system: both hSyn-DIO-hM3Dq-mCherry and diluted CaMKIIa-Cre (10^9^ gc/mL) adeno-associated viruses (AAVs) were mixed and infused bilaterally into the IC according to the brain atlas. Immunohistochemical studies with an antibody against mCherry detected the expression of this viral gene in a region about 1 mm away from the injection sites in the IC. These mice were then subjected to CTA (Fig. 1a). To increase the neuronal activity in a subset of IC neurons, 2 mg/kg CNO was systemically administrated 30 min before CS and US presentations. A memory retrieval test was implemented 1 day following the conditioning in the absence of CNO.

First, we analyzed whether hM3Dq-positive (hM3Dq+) neurons are preferentially activated by conditioning with CNO administration. To visualize these neurons, we used the c-fos protein. The expression of c-fos is rapidly and transiently induced by neuronal activity with the elevation of intracellular Ca^2+^ concentration. The expression of c-fos protein in hM3Dq + neurons was significantly higher in the CNO group than in the saline group (Fig. 1b and c; c-fos+/hM3Dq + neurons (%), *N* = 3 in each group: saline group, 3.0% ± 1.7% CNO group, 66.1% ± 4.9%; *p* = 0.0003). The hM3Dq was expressed in a subset of neurons around the injection site (hM3Dq + neurons/DAPI (%), *N* = 3 in each group: saline group, 1.5% ± 0.3%; CNO group, 2.4% ± 0.2%). However, CNO administration did not affect to the total number of c-fos positive (c-fos+) neurons in IC (Fig. 1d; c-fos+/DAPI (%), *N* = 3 in each group: saline group, 5.9% ± 1.6%; CNO group, 6.4% ± 0.9%; *p* = 0.82) and in BLA (Supplementary Fig. 2A; c-fos+/DAPI (%), *N* = 3 in each group: saline group, 8.0% ± 0.7%; CNO group, 7.6% ± 1.4%; *p* = 0.80). This result indicates that neuronal activation is preferentially induced in hM3Dq-positive neurons by CTA conditioning with CNO administration. Subsequently, we tested whether highly excitable neurons during conditioning are preferentially reactivated by CTA memory retrieval. The hM3Dq was bilaterally expressed in a subset of IC neurons (hM3Dq + neurons/DAPI (%): *N* = 7 in each group; saline group, 3.5% ± 0.4%; CNO group, 3.8% ± 0.5%). After the memory retrieval test, we imaged and analyzed the colocalization of c-fos protein in hM3Dq + neurons. Contrary to our prediction, the probability of c-fos expression induced by memory retrieval in hM3Dq + neurons was not different between the CNO and saline administrated groups, though the probability tended to be increased in the CNO group (Fig. 1e and f; c-fos+ /hM3Dq + neurons (%): *N* = 7 in each group; saline group, 9.4% ± 3.6%; CNO group, 16.4% ± 5.6%; *p* = 0.32). The activation of a small population of IC neurons did not affect the total number of c-fos-positive neurons after memory retrieval (Fig. 1g; c-fos+/DAPI (%):*N* = 7 in each group; saline group, 4.5% ± 0.6%; CNO group, 4.7% ± 0.9%; *p* = 0.88), the quantity of saccharine solution consumed during conditioning (Fig. 1h; drinking amount (g): *N* = 13 in each group; saline group, 1.6 g ± 0.1 g; CNO group, 1.4 g ± 0.1 g; *p* = 0.21), and aversion index (Fig. 1i; aversion index(%): *N* = 13 in each group; saline group, 64.6% ± 6.2%; CNO group, 65.4% ± 7.4%; *p* = 0.94). The aversion index in our methods is relatively weaker than other studies. Therefore, we also showed drinking amounts during retrieval test, in which mice avoided drinking saccharine solution (Supplementary Fig. 1A). These data suggest that an increase in the neuronal activity induced by hM3Dq activation with learning only in a subset of IC neurons cannot determine the neurons that are preferentially activated by CTA memory retrieval.

### Activation of BLA neurons involved in a CTA memory retrieval depends on neuronal activity during learning

**Fig. 2:**
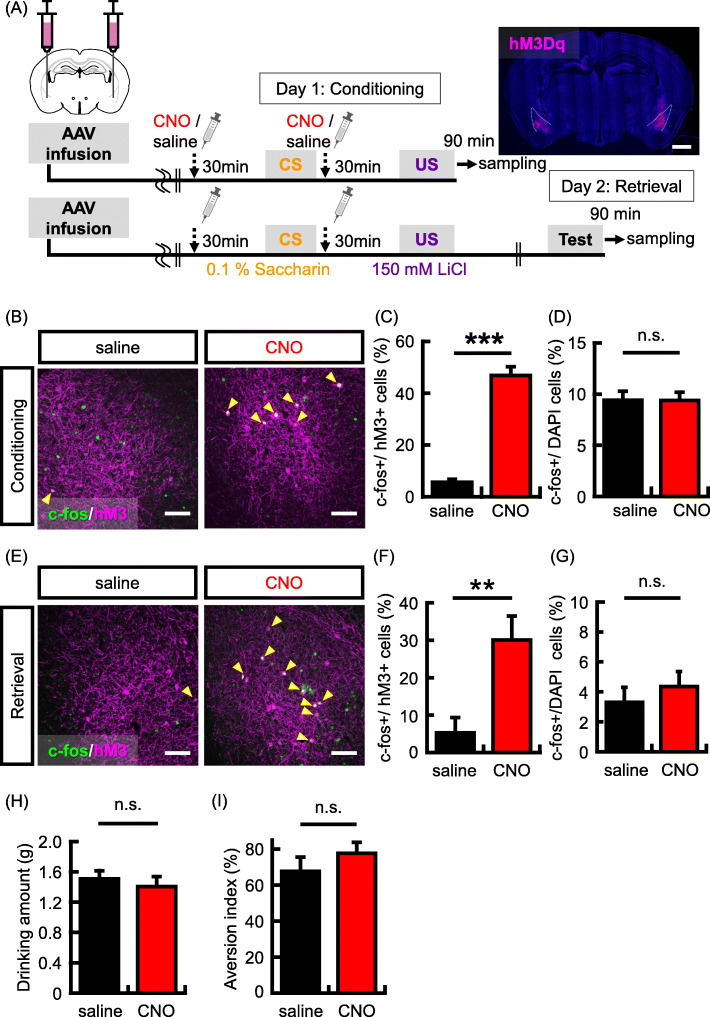
Neural activity regulates taste memory allocation in BLA neurons. **a** Experimental schema. Representative picture shows localized hM3Dq (magenta) expression in the BLA. Scale bar = 1000 um. **b** Representative images showing expression of c-fos (green) following conditioning and hM3Dq (magenta). Yellow arrows indicate double-labeled neurons (c-fos + and hM3Dq+). Scale bar = 100 um.**c-d** Expression probability of expression of c-fos + following conditioning in hM3Dq + neurons (**c**) and DAPI+ cells (**d**) in the BLA (saline group, *N* = 4, CNO group, *N* = 5 animals). **e** Representative images showing the expression of c-fos (green) following memory retrieval test and hM3Dq (magenta). Yellow arrows indicate double-labeled neurons (c-fos + and hM3Dq+). Scale bar = 100 um. **f-g** Probability of occurrence of c-fos + neurons in the hM3Dq + neurons (**f**) and DAPI+ cells (**g**) in the BLA (saline group, *N* = 7, CNO group, *N* = 8 animals). **h** Data showing the mean quantity of saccharine solution consumed during the conditioning. **i** Data showing the aversion index in the memory retrieval test (saline group, *N* = 9 in each group). Data are presented as mean ± SEM; **p < 0.01 and ***p < 0.001 (saline-injected mice, black columns; CNO-injected mice, red columns)

Previous studies showed that fear memory is preferentially recruited into highly excitable neurons in the amygdala [33, 37]. As in the case of the previous study, we used hM3Dq to manipulate neuronal activity. Similar to the IC-related experiment, hM3Dq was bilaterally expressed in a subset of BLA neurons (Fig. 2a). First, we investigated whether hM3Dq + BLA neurons were preferentially activated by conditioning with CNO administration. The c-fos expression in hM3Dq + neurons was significantly higher in the CNO group than in the saline group (Fig. 2b and c; c-fos+/hM3Dq + neurons (%): saline group, 5.6% ± 1.2%, *N* = 4; CNO group, 46.8% ± 3.4%, *N* = 5; *p* = 0.00002). CNO administration had no effect on the total number of c-fos + neurons in either the BLA (Fig. 2d; c-fos+/DAPI (%): saline group, 9.4% ± 0.9%; CNO group, 9.4% ± 0.8%; *p* = 0.98) or in the IC (Supplementary Fig. 2B; c-fos+/DAPI (%): saline group, 6.6% ± 0.8%, *N* = 4; CNO group, 5.6% ± 0.9%. *N* = 5; *p* = 0.42). This result indicates that neuronal activation is preferentially induced in hM3Dq + BLA neurons by CTA conditioning with CNO administration. Next, we tested whether the recruitment of taste memory is also regulated by neuronal activity in the BLA. Again, hM3Dq was bilaterally expressed in a subset of BLA neurons (hM3Dq + neurons/DAPI (%): saline group, 2.1% ± 0.5%, *N* = 7; CNO group, 2.9% ± 0.4, *N* = 8), and the activity of hM3Dq + neurons was increased by the administration of CNO during conditioning (Fig. 2a). Memory retrieval test was implemented 1 day after learning, and brain samples were harvested 90 min after the test. Subsequently, we analyzed the colocalization of c-fos protein in hM3Dq + neurons. Consistent with memory allocation in fear learning, the probability of c-fos expression in hM3Dq + neurons was significantly higher in the CNO group than in the saline group (Fig. 2e and f; c-fos+/hM3Dq + neurons (%): saline group, 5.3% ± 4.1%, *N* = 7; CNO group, 30.1% ± 6.4%, *N* = 8; *p* = 0.008). However, the total number of c-fos + neurons was not different between the saline and CNO groups (Fig. 2g; c-fos+ /DAPI (%): saline group, 3.3% ± 0.8%, *N* = 7; CNO group, 4.4% ± 0.9%, *N* = 8; *p* = 0.41). For the behavioral performance, increased neuronal activity in a subset of BLA neurons did not affect to the quantity of saccharine solution consumed during conditioning (Fig. 2h; drinking amount (g), *N* = 9 in each group: saline group, 1.5 g ± 0.1 g; CNO group, 1.4 g ± 0.1 g; *p* = 0.56) and aversion index (Fig. 2i; aversion index (%), *N* = 9 in each group: saline group, 67.6% ± 7.9%; CNO group, 77.6% ± 6.0%; *p* = 0.33). We confirmed that mice avoided drinking saccharine solution in the retrieval test (Supplementary Fig. 1B). These data suggest that, during learning, an increase in the neuronal activity by hM3Dq in a subset of BLA neurons can determine the neurons that are preferentially activated by CTA memory retrieval. In the amygdala, the cellular mechanism of memory allocation is shared by the fear and taste memory formation.

### Functional connectivity between the insular cortex and the basolateral amygdala is changed by the formation of stronger conditioned taste memory

**Fig. 3:**
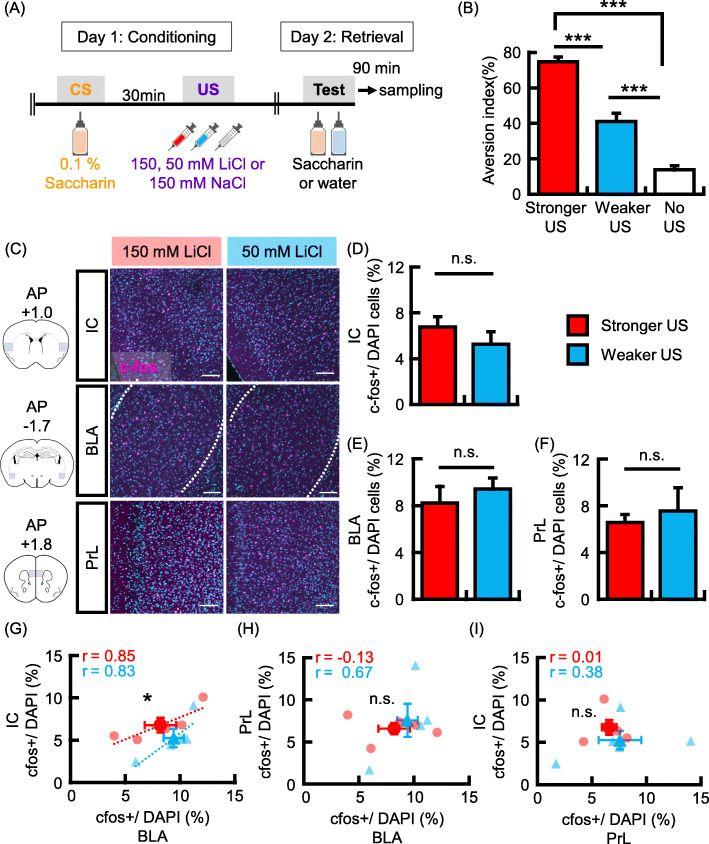
Functional connectivity between the IC and BLA is increased in stronger US conditioning. **a** Experimental schema. **b** Data showing the aversion index in the memory retrieval test (Stronger US, *N* = 14; Weaker US, *N* = 14; No US, *N* = 13). **c** Left panels show schema of representative coronal sections at each AP position (AP = + 1.0 mm; IC, AP = − 1.7 mm; BLA, AP = + 1.8 mm; PrL). Analyzed areas are depicted by a red line. Representative images showing c-fos (magenta) after memory retrieval test. Scale bar = 100 μm. **d-f** Probability of expression of c-fos in DAPI+ cells in the IC (**d**), BLA (**e**) and PrL (**f**) (*N* = 5 in each group). **g-i** Probabilities of occurrence of c-fos + neurons in the IC versus BLA (**g**), BLA versus PrL (**h**), and IC versus PrL (**i**) are plotted for each mouse. The r represents correlation coefficient. Data are represented as mean ± SEM; *p < 0.05 and ***p < 0.001 (Stronger US, red columns and circles; Weaker US group, blue columns and triangles)

Our results suggest that memory allocation in the IC could be regulated by different cellular mechanisms associated with the BLA. The IC and BLA are reciprocally connected, and these interconnections are important for the retention of CTA memory [17]. To know the interaction between IC and BLA, we analyzed the ratio (%) of c-fos + neurons in three brain areas, IC, BLA, and prelimbic cortex (PrL) and the correlations of the ratios between two different brain areas during stronger and weaker US- associated learning. A previous study has demonstrated the involvement of the PrL in CTA acquisition [41]. The novel saccharine taste (CS) was associated with different intensities of aversive experience depending on the administration of 150 mM lithium chloride (LiCl; Stronger US), 50 mM LiCl (Weaker US), or 150 mM NaCl (no US) in the stronger, weaker, or no US-associated learning groups, respectively. Memory retrieval test was implemented 1 day after conditioning (Fig. 3a). Aversion index was significantly higher in the stronger-US associated learning group than in the weaker and no US-associated learning groups, and aversion index of the weaker US group was significantly higher than that of the no US group (Fig. 3b; aversion index: Stronger US group, 74.7% ± 2.7%, *N* = 14; Weaker US group, 41.0% ± 4.7%, *N* = 14; no US group, 13.7% ± 2.3%, *N* = 13; one-way analysis of variance (ANOVA); F_(2,38)_ = 77.9, *p* = 3.6 × 10^− 14^; Tukey-Kramer test, Stronger vs. Weaker US group, *p* = 7.1 × 10^− 8^; Stronger vs. No US group, *p* = 1.4 × 10^− 13^; Weaker vs. No US group, *p* = 6.8 × 10^− 6^). These results suggest that the association between saccharine taste and aversive experience developed in both the stronger and weaker US-associated learning groups, and the strength of the association was dependent on the concentration of LiCl.

To test whether the strength of CS-US association is related to the number of neurons activated by memory retrieval, we analyzed the number of c-fos + neurons in the IC, BLA, and PrL, after the retrieval test. In the three brain areas, the number of c-fos-positive neurons showed no differences between the stronger and weaker US-associated learning groups (Fig. 3c-f; c-fos+ /DAPI (%), *N* = 5 in each group: [D]; IC, stronger-learning group, 6.8% ± 0.9%; weaker-learning group, 5.3% ± 1.1%; *p* = 0.31; [E]; BLA, stronger-learning group, 8.2% ± 1.4%; weaker-learning group, 9.4% ± 0.9%; *p* = 0.50; [F]; PrL, stronger-learning group, 6.6% ± 0.7%; weaker-learning group, 7.6% ± 2.0%; *p* = 0.65). These results suggest that the strength of CTA memory recall does not depend on how many neurons are activated in these brain areas. Subsequently, we analyzed the correlation of c-fos + neurons between brain areas. The correlation coefficients for the number of c-fos-positive neurons between the IC and BLA were 0.85 and 0.83 in stronger and weaker US-associated learning groups, respectively (Fig. 3g). However, such as strong correlation was not observed between the BLA-PrL (Fig. 3h; stronger-learning, *r* = − 0.13; weaker-learning, *r* = 0.67) and IC-PrL (Fig. 3i; stronger-learning, *r* = 0.01; weaker-learning, *r* = 0.38). These results suggest that IC and BLA neurons could be coactivated during memory recall. Subsequently, we analyzed the differences in the number of c-fos + IC neurons correlating to that of c-fos + BLA neurons between the stronger and weaker US-associated learning groups. The number of c-fos-positive neurons in IC correlating to that in BLA was significantly higher in the stronger US-associated learning group than in the weaker US-associated learning group (Fig. 3g, *p* = 0.04). These results suggest that the interaction of cell assembly between the IC and BLA is correlated with the strength of memory retrieval.

### BLA neurons projecting on the IC are preferentially activated by CTA memory retrieval

To test which neural circuit is related to CTA memory retrieval, we used the retrograde neuronal tracer cholera toxin B subunit (CTB) along with c-fos protein imaging. When Alexa 555 labeled CTB was locally infused into the BLA or IC (Supplementary Fig. 3A), and CTB-positive (CTB+) neurons were observed in the IC or BLA, respectively (Supplementary Fig. 3A and B). We analyzed c-fos expression on CTB+ and CTB- excitatory neurons following memory retrieval. CaMKIIa protein was used as a marker of excitatory neurons, and the probability of c-fos expression on CTB+ excitatory neurons was compared with that on CTB- excitatory neurons. There was no difference between numbers of c-fos+/CTB+ and c-fos+/CTB- cells in IC, when CTB was infused into the BLA (Supplementary Fig. 3B and C; *N* = 6, c-fos+/CTB+ neurons (%), 4.4% ± 1.6%; c-fos+/CTB- neurons (%), 5.3% ± 1.0%; *p* = 0.67). On the contrary, the probability of c-fos expression on CTB+ BLA neurons was significantly higher than that on CTB- neurons (Supplementary Fig. 3B and D; *N* = 6, c-fos+/CTB + neurons (%), 9.0% ± 1.2%; c-fos+/CTB- neurons (%), 4.3% ± 0.6%; *p* = 0.005), when CTB was infused into the IC. We confirmed that CTA memory formation was not impaired by the infusion of CTB into BLA (Supplementary Fig. 3E; drinking amount (g), *N* = 6, saccharine solution, 0.3 g ± 0.1 g; tap water, 1.2 g ± 0.1 g; *p* = 0.00008) and IC (Supplementary Fig. 3F; drinking amount (g), *N* = 6, saccharine solution, 0.5 g ± 0.1 g; tap water, 1.0 g ± 0.2 g; *p* = 0.03). These results indicate that neuronal populations in the BLA which projecting to the IC would be preferentially activated by CTA memory retrieval in comparison to other populations. However, additional experiments such as the analysis of no retrieval group is important to further show that BLA neurons projecting to IC are preferentially incorporated into CTA memory trace. Additionally, further investigations are warranted to show the contribution of BLA and IC projecting circuits, their functional significance, and contribution of other circuits to memory formation.

### IC neurons reciprocally connected with the BLA are preferentially activated by CTA memory retrieval

**Fig. 4:**
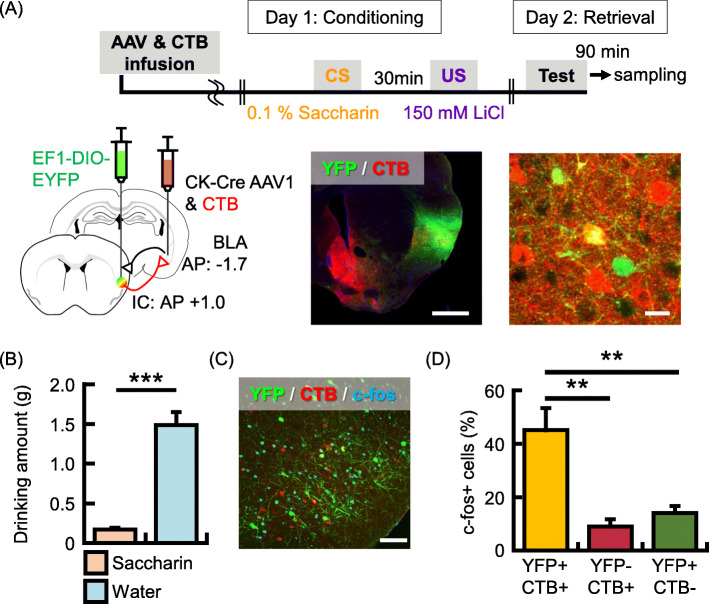
IC neurons reciprocally connected with the BLA are preferentially activated by CTA memory retrieval. **a** Experimental schema (top panel). CTB and AAV_1_-hSyn-Cre injection into the BLA and AAV_1_-Ef1a-DIO EYFP injection into the IC (bottom left panel). Representative image showing coronal section of the IC with CTB (red) and YFP (green) labeled cells. Scale bars = 1000 um (bottom middle). Magnified representative image showing double-positive (yellow, reciprocally connected), YFP+/CTB- (BLA-to-IC only) and YFP−/CTB+ (IC-to-BLA only) IC neurons (bottom right). Scale bar = 20 um. **b** Data showing the mean quantity of saccharine solution (orange column) and water (blue column) consumed in the retrieval test (*N* = 5 animals). **c** Representative image showing expression of c-fos (cyan) following memory retrieval test, CTB (red) and YFP (green). Scale bar = 100 um. **d** Probability of occurrence of cells expressing c-fos after retrieval test over double-positive (yellow column), YFP+ ∩ CTB- (red column) and YFP- ∩ CTB+ (green column) cells in the IC. Data are represented as mean ± SEM; **p < 0.01 and ***p < 0.001

To examine further a neural circuit related to CTA memory retrieval, we used the AAV-mediated anterograde transsynaptic tagging [42] combined with CTB and c-fos imaging. In this experiment, Alexa 647 labeled CTB and hSyn-Cre AAV1 were locally infused into the BLA, and the Cre-dependent YFP AAV was locally infused into the IC (Fig. 4a). CTB-, YFP- and double-positive cells were observed in IC (Fig. 4a, *N* = 5, CTB+ cells/DAPI (%), 3.6% ± 1.0%; YFP+ cells/DAPI (%), 3.7% ± 0.9%; double-positive cells/DAPI (%), 0.5% ± 0.2%). CTA was performed 3 weeks after the surgery, and mice were perfused for brain sampling after the memory retrieval test (Fig. 4b; drinking amount (g), *N* = 5, saccharine solution, 0.2 g ± 0.02 g; tap water, 1.5 g ± 0.2 g; *p* = 0.00005). Subsequently, we analyzed the probability of c-fos expression on IC neurons that have a different neural connection with BLA neurons. Interestingly, the probability of c-fos expression in double-positive neurons was significantly higher than that in solely CTB+ and YFP+ cells (Fig. 4c and d, *N* = 5, c-fos+/CTB+ ∩ YFP+ [reciprocal] (%), 45.1% ± 8.3%; c-fos+/CTB- ∩ YFP+ [IC-from-BLA] (%), 14.0% ± 2.7%; c-fos+/CTB+ ∩ YFP- [IC-to-BLA] (%), 9.0% ± 2.7%; F_(2, 12)_ = 13.8, *p* = 0.0008; Tukey-Kramer test, [reciprocal] vs. [IC-from-BLA], *p* = 0.003; [reciprocal] vs. [IC-to-BLA], *p* = 0.001; [IC-from-BLA] vs. [IC-to-BLA], *p* = 0.78). Collectively, these results suggest that IC neurons reciprocally connected with the BLA are preferentially activated by CTA memory retrieval.

### Co-activation of the IC and BLA determines the IC neurons that are activated by CTA memory retrieval

**Fig. 5:**
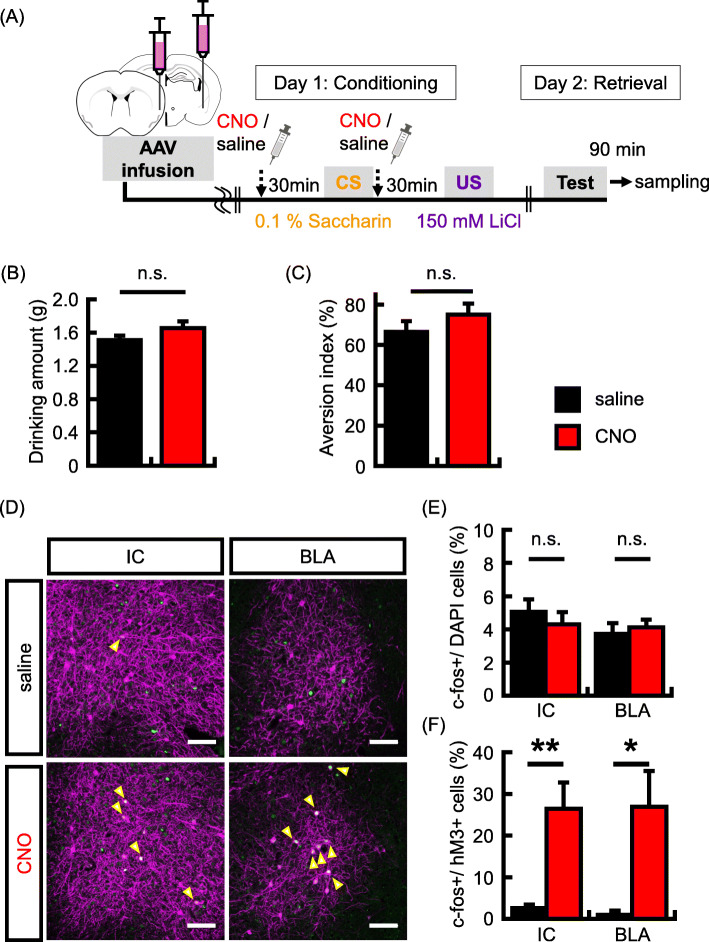
Co-activation of IC and BLA neurons regulates taste memory allocation in the IC. **a** Experimental schema. **b** Data showing the mean quantity of saccharine solution consumed during the conditioning. **c** Data showing the aversion index in memory retrieval test (saline group, *N* = 16, CNO group, *N* = 17 animals). **d** Representative images showing expression of c-fos (green) after memory retrieval test and that of hM3Dq (magenta). Yellow arrows indicate double-labeled neurons (c-fos + and hM3Dq+). Scale bars = 100 um. **e-f** Probability of expression of c-fos in DAPI cells (**e**) and in the hM3Dq + neurons (**f**) in the IC and BLA (IC, saline group, *N* = 5, CNO group, *N* = 6 animals; BLA, saline group, *N* = 5, CNO group, *N* = 6 animals). Data are represented as mean ± SEM; *p < 0.05 and **p < 0.01 (saline-injected mice, black columns; CNO-injected mice, red columns)

Our c-fos imaging experiments suggest that IC neurons activated by CTA memory retrieval could be regulated by the interaction between the IC and BLA. Subsequently, we tested whether activating both the IC and BLA neurons during conditioning changes the IC neurons that are activated by memory retrieval. hM3Dq was expressed in a subset of IC and BLA neurons (hM3Dq + neurons/DAPI (%): IC, saline group, 2.3% ± 0.3%, *N* = 5; CNO group, 2.2% ± 0.3%, *N* = 6; BLA, saline group, 1.2% ± 0.3%, *N* = 5; CNO group, 1.8% ± 0.1%, *N* = 6), and neuronal activity in the hM3Dq neurons were induced by the administration of CNO during conditioning (Fig. 5a). Memory retrieval test was implemented 1 day after conditioning, and mice were perfused for brain sampling, 90 min following the retrieval test. For the CTA memory formation, increased neuronal activity in a subset of IC and BLA neurons did not affect the quantity of saccharine solution consumed during conditioning (Fig. 5b; drinking amount (g): saline group, 1.5 g ± 0.1 g, *N* = 16; CNO group, 1.7 g ± 0.1 g, *N* = 17; *p* = 0.15) and aversion index (Fig. 5c; aversion index (%): saline group, 66.4% ± 5.3%, *N* = 16; CNO group, 75.1% ± 5.4%, *N* = 17; *p* = 0.26). We confirmed that mice avoided drinking saccharine solution in the retrieval test (Supplementary Fig. 1C). In the imaging analysis, the total number of c-fos + neurons was not different between the saline and CNO groups (Fig. 5e; c-fos+ /DAPI (%): IC, saline group, 5.1% ± 0.8%, *N* = 5; CNO group, 4.3% ± 0.7%, *N* = 6, *p* = 0.50; BLA, saline group, 3.7% ± 0.7%, *N* = 5; CNO group, 4.1% ± 0.5%, *N* = 6; *p* = 0.63). Subsequently, we analyzed the colocalization of c-fos protein in hM3Dq + neurons. Interestingly, unlike the individually increased neuronal activity in the IC using hM3Dq alone, the probability of c-fos expression in hM3Dq + neurons in IC was significantly higher in the CNO group than in the saline group (Fig. 5d and f; c-fos+/hM3Dq + neurons (%): saline group, 2.6% ± 0.8%, *N* = 5; CNO group, 26.5% ± 6.3%, *N* = 6; *p* = 0.007). In the BLA, consistent with the above results, the corresponding probability was significantly higher in the CNO group than in the saline group (Fig. 5d and f; c-fos+/hM3Dq + neurons (%): saline group, 1.0% ± 1.0%, *N* = 5; CNO group, 26.9% ± 8.6%, *N* = 6; *p* = 0.02). To test whether CNO administration affects c-fos expression in IC and BLA neurons, mCherry was expressed in a subset of IC and BLA neurons and analyzed for c-fos expression. CNO was administrated during conditioning. Memory retrieval test was implemented 1 day after conditioning, and mice were perfused for brain sampling, 90 min following the retrieval test. For the CTA memory formation, CNO administration did not affect the quantity of saccharine solution consumed during conditioning (Supplementary Fig. 4A; drinking amount (g): *N* = 6 in each group: saline group, 1.6 g ± 0.1 g; CNO group, 1.5 g ± 0.1 g; *p* = 0.28) and aversion index (Supplementary Fig. 4B; aversion index (%): *N* = 6 in each group: saline group, 83.2% ± 5.4%; CNO group, 80.2% ± 4.9%; *p* = 0.70). In imaging analysis, the total number of c-fos + neurons was not different between the saline and CNO groups (Supplementary Fig. 4C; c-fos+ /DAPI (%): *N* = 5 in each group: IC, saline group, 4.5% ± 0.5%; CNO group, 4.7% ± 1.4%, *p* = 0.89; BLA, saline group, 5.6% ± 0.7%; CNO group, 4.6% ± 0.8%, *p* = 0.36). The probability of c-fos expression in hM3Dq + neurons showed no differences between the IC and BLA (Supplementary Fig. 4D; c-fos+ / mCherry+ neurons (%): *N* = 5 in each group: IC, saline group, 6.2% ± 0.7%; CNO group, 5.8% ± 0.9%, *p* = 0.75; BLA, saline group, 7.3% ± 1.1%; CNO group, 6.0% ± 1.2%, *p* = 0.46). These results suggest that the coactivation of the IC and BLA during conditioning determines the IC neurons that are activated by CTA memory retrieval.

## Discussion

In the present study, we used the hM3Dq receptor to manipulate the neuronal activity in a subset of IC and/or BLA neurons during CTA conditioning. When the hM3Dq receptor was activated using CNO alone in a subset of IC neurons, c-fos expression following memory retrieval was not preferentially observed in hM3Dq + neurons. Interestingly, the probability of c-fos expression in hM3Dq + IC neurons after retrieval was significantly increased when the both IC and BLA neurons were activated during conditioning using the hM3Dq system. In the BLA, c-fos expression was preferentially induced in hM3Dq + neurons when neuronal activity was increased only in the BLA and in both the BLA and IC during conditioning. The functional connectivity between the IC and BLA was more strengthened under stronger-US conditioning than under weaker-US conditioning. We also examined which neuronal circuit that relates a CTA memory retrieval, using retrograde and anterograde transsynaptic tracers. Our results show that memory allocation in IC neurons is regulated by interaction with BLA neurons.

In previous studies, hM3Dq receptors and SFO were used to manipulate neuronal activity in the amygdala, and these studies showed that neuronal activity at the time of learning regulates fear memory allocation [33, 37]. Consistent with these studies, manipulation of BLA neurons using hM3Dq receptors helped determine the BLA neurons that are activated by memory retrieval. Previous studies showed that CREB activity is regulated by previous learning and that increased CREB activity results in an increased neuronal activity [31, 38]. In both fear conditioning and CTA, memory allocation in the amygdala is regulated by CREB [29–33]. Therefore, molecular and cellular mechanisms of memory allocation are shared in a different type of memory formation in the amygdala.

We previously showed that CREB regulates memory allocation in the IC; using the neuronal-inactivation system, we showed that CTA memory retrieval was impaired by selectively silencing neurons with virally transduced CREB (vCREB) [28]. Additionally, we also showed that vCREB+ IC neurons are preferentially activated after memory retrieval using the expression of the immediate early gene arc [28]. In the previous study, CREB level was manipulated only in a subset of IC neurons. On the contrary, in this study, IC neurons that were activated during conditioning, were not preferentially activated after memory retrieval when neuronal activity was manipulated in the IC alone. One possible explanation for this is that the prolonged expression of CREB in the IC affects its connectivity with BLA because CREB regulates the formation of neuronal circuit [43, 44] and because taste memory could be allocated into a subset of IC-BLA circuit in which synaptic connection is strengthened by CREB. In the present study, we observed that IC neurons reciprocally connected with the BLA were preferentially activated after memory retrieval. These results indicate that predetermined circuit connection could be one basis of the memory allocation, and non-selective hM3Dq activation only in the IC could induce a noise to interfere engram formation. On the contrary, co-activation of the IC and BLA using hM3Dq system during conditioning biased c-fos expression after memory retrieval in IC neurons. Taken together, our findings suggest that the allocation of taste memory to a subset of IC neurons is regulated by their interaction with the BLA. In this study, we manipulated a small subset of neurons to histologically analyze whether c-fos expression following memory retrieval is induced in these neurons, and then could not behaviorally analyze the effect on the retrieval. Therefore, further studies are warranted to functionally show that those neurons are preferentially incorporated into neurocircuits encoding CTA such as silencing reciprocally connected engram cells in BLA and IC, and how co-activation of the IC and BLA affects the memory retrieval.

Based on our findings, we suggest that the interactions and connections between IC and BLA neurons are important to determine cell assembly for CTA memory retrieval. Additionally, we showed that the number of c-fos + neurons in the IC that correlates to that of the BLA was higher in the stronger-US conditioning group than in the weaker-US conditioning group. This result suggests that the functional connectivity between the IC and BLA could be important for CTA memory formation. A previous electrophysiological study showed that CTA learning enhances the functional connectivity between the IC and BLA [24]. In the IC, LTP is induced by the high frequency stimulation of BLA, which modulates CTA memory formation [18–22]. Recently, it is shown that IC-to-BLA projecting neurons are an essential components of the CTA acquisition and memory retrieval [26]. Consistent with these findings, c-fos expression after memory retrieval was preferentially observed in the IC neurons reciprocally connected to the BLA in our experiment. In the hippocampus, it was shown that enhanced structural and functional connectivity between engram cells in CA1 and CA3 forms the synaptic correlate for memory formation [45]. Coordinated activation of cell assembly in the IC and BLA during CTA learning could strengthen the synaptic connection between engram cells in these two brain areas. In this study, manipulation of neuronal activity in a random population of IC and BLA neurons could partially mimic this process, and subsequently, coactivated IC and BLA neurons during learning could be preferentially reactivated by memory retrieval. Since we manipulated random neuronal populations using hM3Dq-DREADD systems, in which activation is not physiological, in future studies manipulating physiologically activated neurons will also be necessary to elucidate the circuit mechanism of memory allocation. Furthermore, a recent study had shown that LTD in BLA to IC pathway is occluded by CTA learning and modulates sucrose preference [46]. Further studies are necessary to address which type of synaptic plasticity is induced in engram cells and how BLA modulates IC neurons in CTA.

The IC is the primary gustatory cortex and processes taste information [1–3]. Interestingly, firing rates in response to a taste in IC neurons is changed by inactivation of BLA [47]. By CTA learning, cellular representation to taste valence is changed from appetitive to aversive in IC neurons [25, 48]. Furthermore, BLA activation enhances plasticity in IC and CTA memory formation [20, 49]. Perhaps taste information associated with emotional valence is encoded in the circuit-level, and coordination of memory allocation across brain regions regulates the encoding of multimodal information.

In summary, the findings presented herein show that the functional interaction between the IC and BLA plays an important role in the allocation of CTA memory.

## Materials and methods

### Animals

All experimental protocols were evaluated and approved by the Regulation for Animal Research at Tokyo University of Science. All experiments were conducted in accordance with the Regulations for Animal Research at the Tokyo University Science. Mice were individually housed in a 12-h (7:30 am to 7:30 pm) light/dark cycle with access to food and water ad libitum. We used 3 to 6 month-old C57BL/6 J male mice (SLC Japan).

### Virus vectors

AAV_8_- hSyn-DIO-hM3D(Gq)-mCherry (2.5 × 10^13^ genome copy/mL, #44361-AAV8), AAV_8_- hSyn-DIO- mCherry (1.0 × 10^13^ genome copy/mL, # 50459-AAV8) and AAV_1_-Ef1a-DIO EYFP (2.2 × 10^13^ genome copy/mL, #27056-AAV1) were purchased from Addgene. AAV_8_- CaMKIIa-mCherry-Cre (4.7 × 10^12^ genome copy/mL) was purchased from the Vector Core at the University of North Carolina at Chapel Hill. AAV_1_- hSyn- Cre (3.3 × 10^13^ genome copy/mL) was purchased from the University of Pennsylvania Vector Core.

### Stereotactic surgery

The mice were anesthetized with pentobarbital (80 mg/kg of body weight by intraperitoneal injection), and administered carprofen (5 mg/kg of body weight; subcutaneous injection); and the fully anesthetized mice were placed in a stereotactic apparatus (Narishige, Japan). A 2-mm diameter craniotomy was performed above the IC cortex. A 200 nL virus solution were infused at a rate of 0.1 uL/min using a Hamilton syringe through a glass micropipette at the following coordinates, as described in the mouse brain atlas [50]: relative to bregma (mm): IC, anteroposterior axis (AP): + 1.0, mediolateral axis (ML): ± 3.6, and dorsoventral axis (DV): + 2.5 from dura mater; BLA, AP: -1.7, ML: ± 3.3, and DV: − 5.0 from dura mater. A glass capillary was left in place for an additional 10 min. In the experiments involving the express of hM3Dq in a subset of neurons (Figs. 1, 2 and 5), diluted AAV_8_-CaMKIIa-mCherry-Cre (10^9^ genome copy/mL) and AAV_8_- hSyn-DIO-hM3D(Gq)-mCherry or AAV_8_- hSyn-DIO- mCherry were infused into target brain areas. AAV solution was bilaterally infused in the experiment of Figs. 1 and 2. In the other experiments, AAV solution was unilaterally infused. In the retrograde labeling experiment, 100 nL of CTB-Alexa555 (0.5% wt/vol) was unilaterally injected into the BLA or IC (Supplementary Fig. 3). In the reciprocal labeling experiment, 200 nL mixture of CTB-Alexa647 (0.45% wt/vol) and hSyn-Cre AAV (10^12^ genome copy/mL) was infused into the BLA, and 200 nl solution of Ef1a-DIO EYFP was infused into the IC. Behavioral tests were implemented approximately 4 weeks after surgery for the experiment infusing AAVs (Figs. 1, 2, 5 and Supplementary Fig. 4) and 3 weeks after surgery for other experiments infusing CTB and/or AAV (Fig. 4 and Supplementary Fig. 3) after surgery to allow for sufficient expression of genes and recovery of mice.

### Immunohistochemistry and analysis of c-fos positive neurons

Mice were deeply anesthetized with pentobarbital, administered carprofen, and transcardially perfused with 4% (w/v) paraformaldehyde in 0.1 M sodium phosphate buffer, pH 7.4. The brains were excised, postfixed with the same fixative at 4 °C overnight and equilibrated in 30% (w/v) sucrose in phosphate-buffered saline (PBS) as a cryoprotectant. The brains were embedded in O.C.T. compound (Sakura Finetech), and frozen coronal sections (50 μm) were prepared. Free-floating sections were incubated with 0.2% (v/v) Triton X-100 in PBS, and then blocked with 5% (v/v) Donkey serum and 0.2% (v/v) Triton X-100 in PBS. Sections were incubated with primary antibodies against c-fos (1:200; Synaptic Systems; 226,003), mCherry (1:5000; Thermo Fisher Scientific; M11217), GFP (1:1000; Aves Labs Inc.; GFP-1020), and CaMKIIa (1:5000; Thermo Fisher Scientific; M11217) at 4 °C for two nights. Subsequently, sections were incubated with an Alexa488-conjugated anti-rabbit IgG, Alexa546-conjugated anti-rabbit IgG, Alexa546-conjugated anti-rat IgG, Alexa488-conjugated anti-chicken IgY and Alexa647-conjugated anti-mouse IgG (1:1000; Life Technologies). Nuclei were stained using DAPI (Life Technologies). Sections were imaged with a confocal microscope (Olympus; FV1000). Confocal images of the IC and BLA were acquired using a 20× objective lens.

Manual cell counts were performed by an experimenter blinded to the treatment. Small, bright, and uniformly DAPI-stained nuclei from the putative glial cells were not counted. Each brain area was identified according to a mouse brain atlas [50] (IC, AP, around + 0.6, + 0.8, + 1.0, + 1.2, and + 1.4 mm; BLA, AP around − 1.6, − 1.8, and − 2.0 mm; PrL, AP, around + 1.6 and + 1.8 mm). The probability of expression of c-fos + on hM3Dq+, CTB+, CaMKIIa+, YFP+ and/or DAPI+ neurons was calculated for each mouse studied.

### Conditioned taste aversion test

Mice were dehydrated by removal of a water bottle for 24 h before each test. Habituation: the thirsty mice were presented with two bottles of tap water for 5 days (for 60 min on day 1, 45 min on day 2, and 30 min on days 3–5). Body weight and water consumption were monitored daily. If an animal lost more than 20% of their body weight relative to that before habituation, they were not used in the ensuing experiments. Conditioning: on day 6, mice were exposed to CS, free access for 15 min to one bottle containing a saccharine solution (0.1% w/v), followed 30 min later by US, an intraperitoneal (i.p.) injection of lithium chloride LiCl (0.15 M, 2% of body weight). In Fig. 3, 0.05 M LiCl in 0.1 M NaCl and 0.15 M NaCl solution was also used as US for weaker- and no-learning groups, respectively. To prevent dehydration, 7–8 h after training, mice were given free access to water for 15 min. Retrieval test: on day 7, a two-choice test was implemented to determine the acquired aversion to a sweet taste. The mice were presented with two choice, water and 0.1% Saccharine solution, for 15 min. Total consumption of each fluid was measured before and after the test. The aversion index was calculated as (Consumption of water in mL/Total consumption of water and saccharine solution in mL) × 100 (%). To increase the neuronal activity using hM3Dq, 2 mg/kg of CNO (Enzo Life Science, BML-NS105) dissolved in saline was intraperitoneally injected into mice 30 min before CS and US presentation. For the immunohistochemical analysis of c-fos protein, brains were extracted 90 min after the CTA conditioning or retrieval test.

### Statistics

Comparisons of data between two groups were analyzed using two-tailed Student’s t-tests, and multiple-group comparisons were performed using one-way ANOVA. Significant main effects were determined using Tukey-Kramer post hoc tests. In Fig. 3g-i, differences in c-fos + neurons between the stronger- and weaker-learning groups were analyzed using an analysis of covariance (ANCOVA). Differences were considered statistically significant when the probability value was < 0.05.

## Supplementary information

**Additional file 1: Supplementary Figure 1.** Drinking amount of saccharine solution and water during retrieval test in mice used for c-fos analysis. (A-C) Data showing the mean quantity of saccharine solution (orange columns) and water (blue columns) consumed during the retrieval test in M3 into IC (A), M3 into BLA (B) and M3 into IC and BLA groups (C), the results of which corresponds to Figs. 1f-g, 2f-g and 5e-f, respectively. Left and right panels showing saline- and CNO-infused group, respectively (M3 into IC, *N* = 7 animals in each group; M3 into BLA, saline group, *N* = 7, CNO group, *N* = 8 animals; M3 into IC and BLA, saline group, *N* = 5, CNO group, *N* = 6 animals). Data are represented as mean ± SEM; *p < 0.05, **p < 0.01 and ***p < 0.001. **Supplementary Figure 2.** The c-fos expression following conditioning in BLA and IC with hM3Dq activation in IC and BLA, respectively. (A and B) Data showing expression probability of c-fos + following conditioning with hM3Dq activation in IC and BLA on DAPI+ cells in the BLA (A) and the IC (B), respectively (BLA, *N* = 3 animals in each group; IC, saline group, *N* = 4, CNO group, *N* = 5 animals). Data are shown as mean ± SEM. (saline-injected mice, black columns; CNO-injected mice, red columns). **Supplementary Figure 3.** BLA-to-IC projection neurons are preferentially activated by CTA memory retrieval. (A) Experimental schema (top panel). CTB injection into the BLA and representative images showing CTB (magenta). Scale bar = 500 um (bottom left panel). CTB injection into the IC and representative images showing CTB (magenta). Scale bars = 500 um (bottom right panel). (B) Representative images showing expression of c-fos (green) following memory retrieval test, CTB (magenta) and CaMKIIa (red). Yellow arrows indicate double-labeled cells (c-fos + and CTB+). Scale bars = 100 um. (C-D) Probability of expression of c-fos over CTB- (black columns) and CTB+ excitatory neurons (red columns) in the IC (C) and the BLA (D) following retrieval test (IC, *N* = 6; BLA, *N* = 6). (E-F) Data showing the mean quantity of saccharine solution (orange columns) and water (blue columns) consumed during the retrieval test in the IC-labeled (E) and BLA-labeled (F) groups (IC-labeled, *N* = 6, BLA-labeled group, *N* = 6 animals). Data are represented as mean ± SEM; *p < 0.05, **p < 0.01 and ***p < 0.001. **Supplementary Figure 4.** CNO administration does not affect c-fos expression in IC and BLA neurons. CaMKIIa-Cre and hSyn-DIO-mCherry AAV were infused into IC and BLA. (A) Mean saccharine solution consumed during conditioning. (B) Aversion index in memory retrieval test (*N* = 6 animals in each group). (C-D) Probability of c-fos expression in DAPI cells (C) and in mCherry+ neurons (D) in the IC (left) and BLA (right) (*N* = 5 in each group). Data are represented as mean ± SEM (saline-injected mice, black columns; CNO-injected mice, red columns).

## Data Availability

The datasets used and/or analyzed during the current study are available from the corresponding author on reasonable request.

## Abbreviations

AAV: Adeno-associated viruses
AI: Aversion index
ANOVA: Analysis of variance
AP: Anteroposterior axis
CNO: Clozapine-N-oxide
CREB: Cyclic-AMP-response-element-binding
CS: Conditioned stimulus
CTA: Conditioned taste aversion
DREADD: Designer receptor exclusively activated by a designer drug
IC: Insular cortex
LTP: Long-term potentiation
PBS: Phosphate-buffered saline
SFO: Step function opsin
US: Unconditioned stimulus
ANCOVA: Analysis of covariance
BLA: Basolateral amygdala
CTB: Cholera toxin B
DAPI: 4′,6-diamidino-2-phenylindole
PrL: Prelimbic cortex
YFP: Yellow fluorescent protein
