# Supplementary figures and images for: Cortico-amygdala interaction determines the insular cortical neurons involved in taste memory retrieval
Source: Mol Brain. 2020 Jul 28;13:107. doi: 10.1186/s13041-020-00646-w (PMC7385890; doi:10.1186/s13041-020-00646-w)

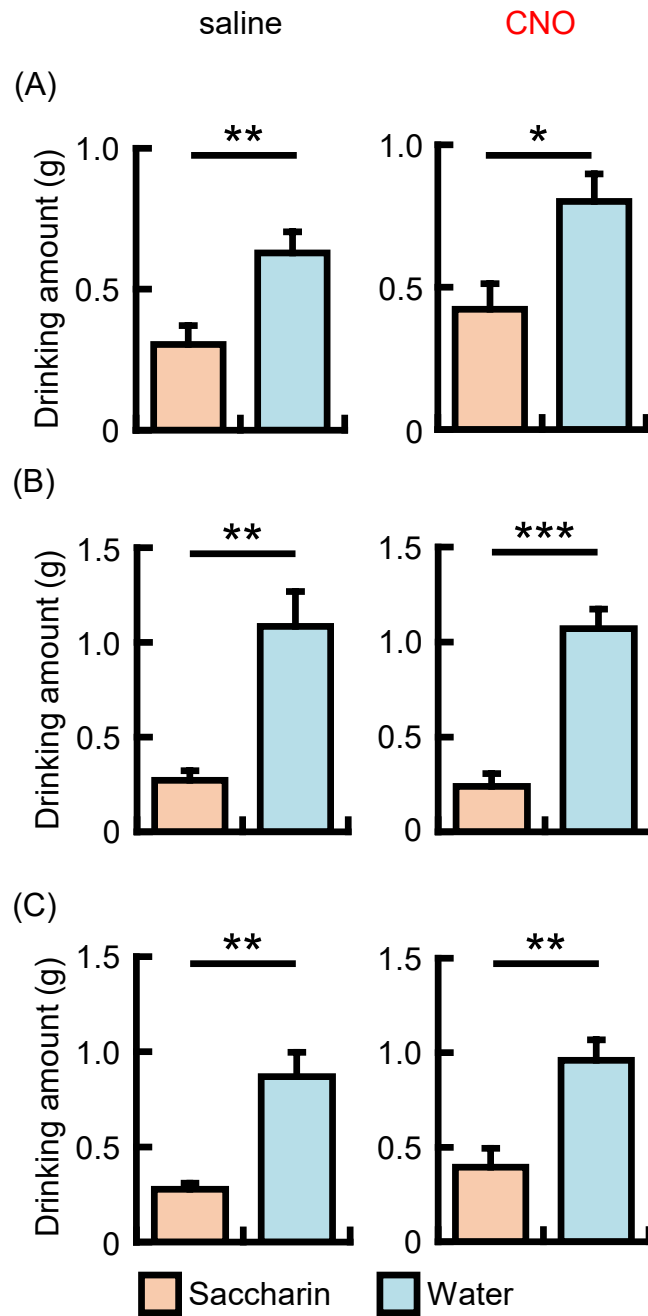

Supplementary Figure. 1

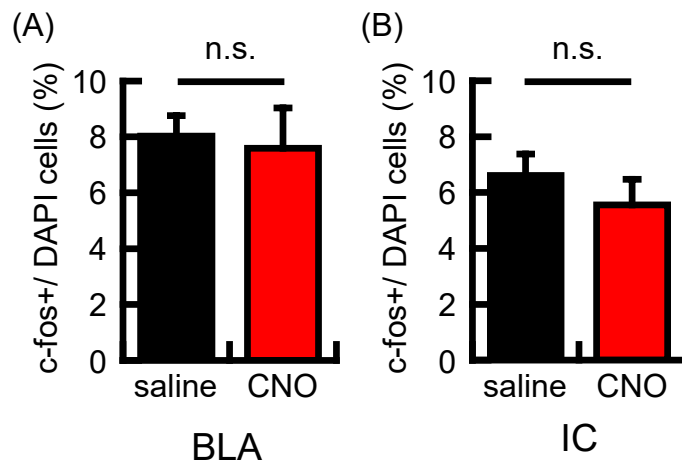

Supplementary Figure. 2

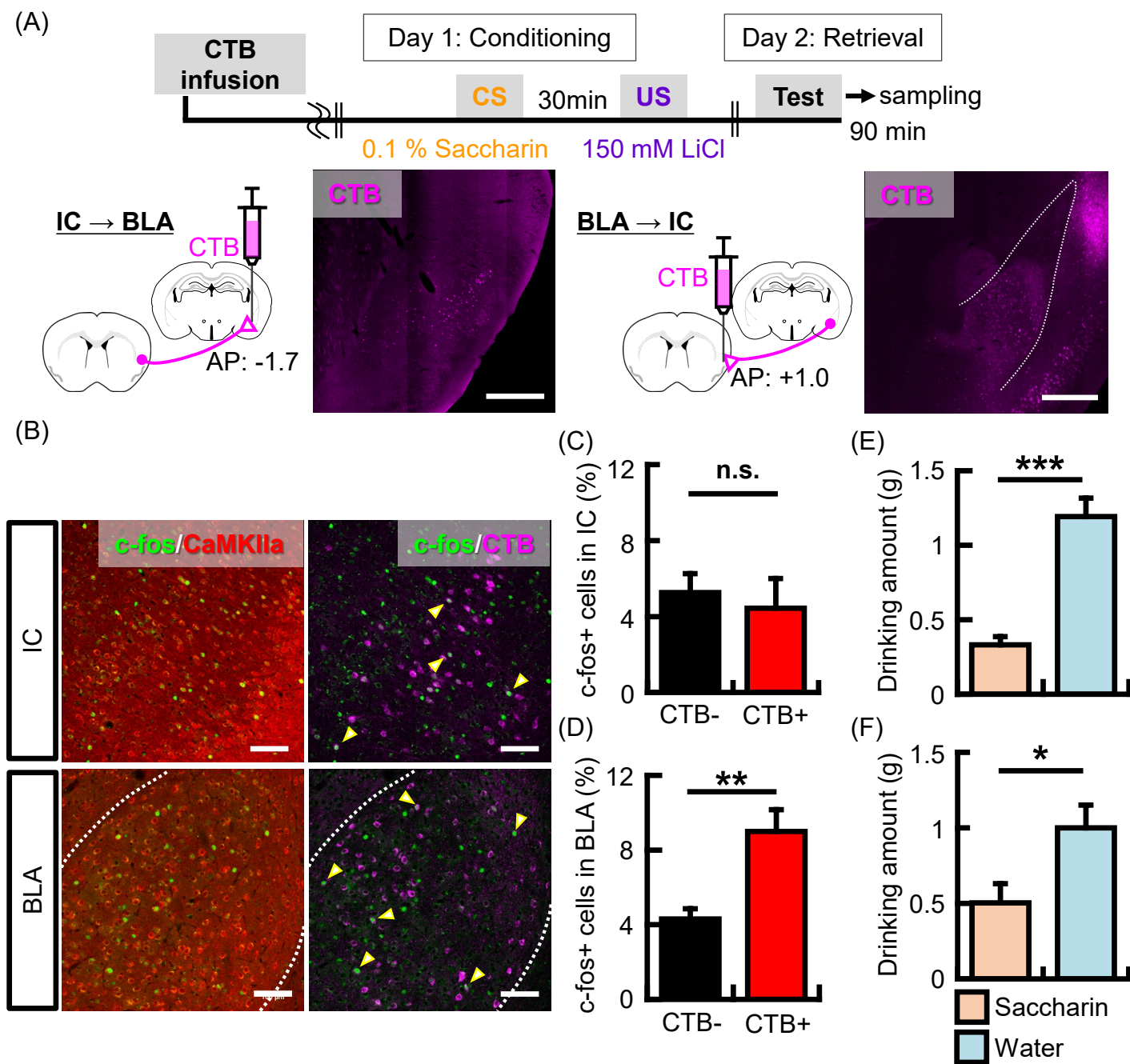

Supplementary Figure. 3

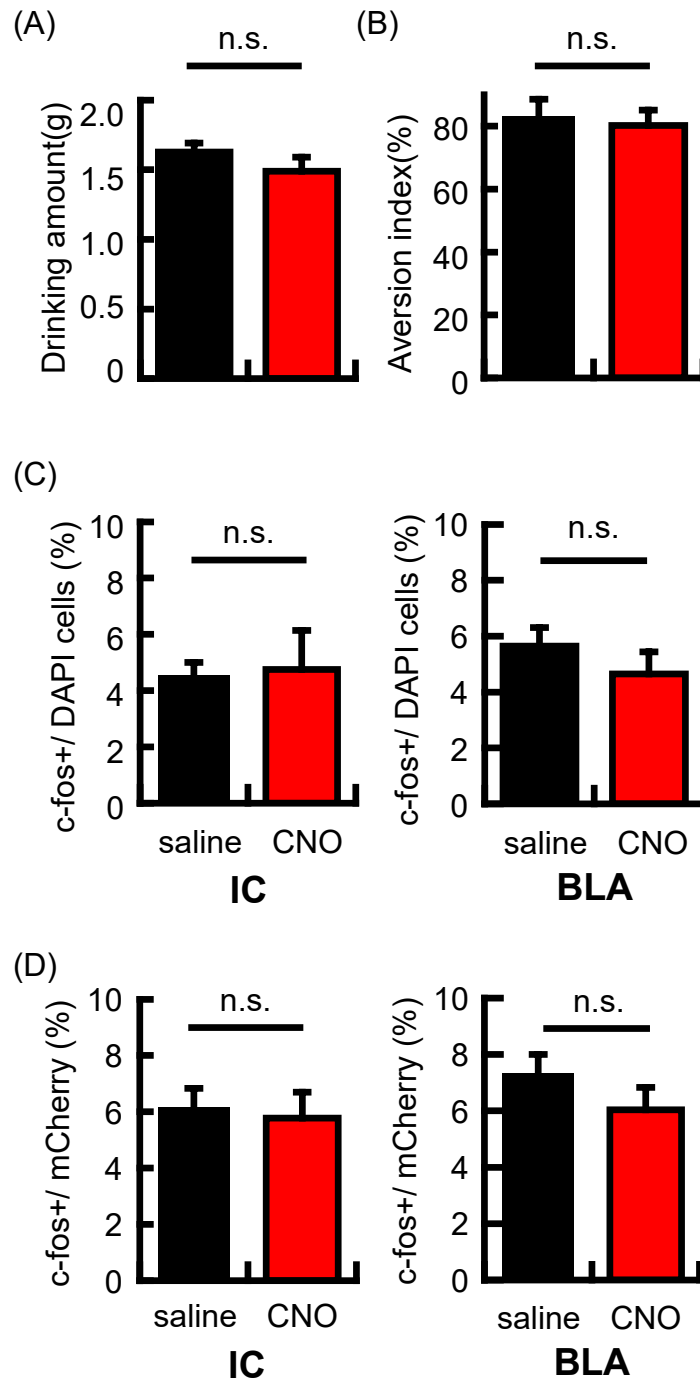

Supplementary Figure. 4

Supplement: Supplementary file 1 — Additional file 1: Supplementary Figure 1. Drinking amount of saccharine solution and water during retrieval test in mice used for c-fos analysis. (A-C) Data showing the mean quantity of saccharine solution (orange columns) and water (blue columns) consumed during the retrieval test in M3 into IC (A), M3 into BLA (B) and M3 into IC and BLA groups (C), the results of which corresponds to Figs. 1f-g, 2f-g and 5e-f, respectively. Left and right panels showing saline- and CNO-infused group, respectively (M3 into IC, N = 7 animals in each group; M3 into BLA, saline group, N = 7, CNO group, N = 8 animals; M3 into IC and BLA, saline group, N = 5, CNO group, N = 6 animals). Data are represented as mean ± SEM; *p < 0.05, **p < 0.01 and ***p < 0.001. Supplementary Figure 2. The c-fos expression following conditioning in BLA and IC with hM3Dq activation in IC and BLA, respectively. (A and B) Data showing expression probability of c-fos + following conditioning with hM3Dq activation in IC and BLA on DAPI+ cells in the BLA (A) and the IC (B), respectively (BLA, N = 3 animals in each group; IC, saline group, N = 4, CNO group, N = 5 animals). Data are shown as mean ± SEM. (saline-injected mice, black columns; CNO-injected mice, red columns). Supplementary Figure 3. BLA-to-IC projection neurons are preferentially activated by CTA memory retrieval. (A) Experimental schema (top panel). CTB injection into the BLA and representative images showing CTB (magenta). Scale bar = 500 um (bottom left panel). CTB injection into the IC and representative images showing CTB (magenta). Scale bars = 500 um (bottom right panel). (B) Representative images showing expression of c-fos (green) following memory retrieval test, CTB (magenta) and CaMKIIa (red). Yellow arrows indicate double-labeled cells (c-fos + and CTB+). Scale bars = 100 um. (C-D) Probability of expression of c-fos over CTB- (black columns) and CTB+ excitatory neurons (red columns) in the IC (C) and the BLA (D) following retrieval test [file 13041_2020_646_MOESM1_ESM.pdf]
